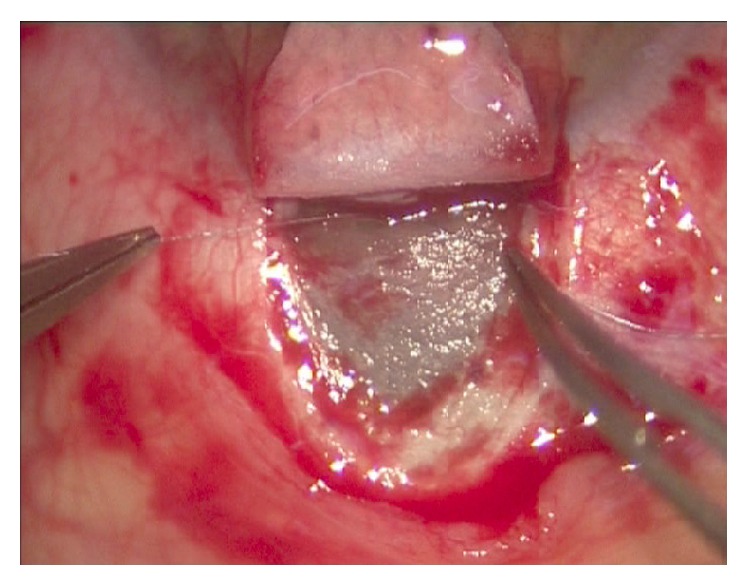# Canaloplasty: Current Value in the Management of Glaucoma

**DOI:** 10.1155/2016/7080475

**Published:** 2016-04-30

**Authors:** Carlo Cagini, Claudia Peruzzi, Tito Fiore, Leopoldo Spadea, Myrta Lippera, Stefano Lippera

**Affiliations:** ^1^Department of Surgery and Biomedical Science, University of Perugia, Ospedale S. Maria della Misericordia, 06156 Perugia, Italy; ^2^Department of Biotechnology and Medical-Surgical Sciences, “Sapienza” University of Rome, 04100 Latina, Italy; ^3^Area Vasta 2 Marche, Ospedale di Fabriano, 60044 Fabriano, Italy

## Abstract

Canaloplasty is a nonpenetrating blebless surgical technique for open-angle glaucoma, in which a flexible microcatheter is inserted within Schlemm's canal for the entire 360 degrees. When the microcatheter exits the opposite end, a 10-0 prolene suture is tied and it is then withdrawn, by pulling microcatheter back through the canal in the opposite direction. Ligation of prolene suture provides tension on the canal and facilitates aqueous outflow. The main advantage of canaloplasty is that this technique avoids the major complications of fistulating surgery related to blebs and hypotony. Currently, canaloplasty is performed in glaucoma patients with early to moderate disease and combination with cataract surgery is a suitable option in patients with clinically significant lens opacities.

## 1. Introduction

Glaucoma is linked to substantial social and economic costs due to its prevalence and deleterious impact on quality of life. Although more than 66 million people worldwide are estimated to be affected, up to 50% of glaucoma cases are undiagnosed [1]. Several studies demonstrated that, lowering intraocular pressure (IOP), the principal risk factor for glaucoma progression reduced the progression rate and efficiently preserved sight [2–4]. Standard glaucoma treatment is drug therapy followed by surgery when optimal disease control is not obtained. Trabeculectomy, the gold standard in glaucoma surgery, drains the aqueous humor from the anterior chamber to the subconjunctival space but is associated with a high rate of complications, that is, hypotony, hyphema, choroidal detachment, suprachoroidal hemorrhage, blebitis, and bleb-associated endophthalmitis [5, 6]. Consequently, innovative surgical techniques, such as viscocanalostomy and canaloplasty, were proposed to control IOP. Canaloplasty may be considered the evolution of viscocanalostomy which was described by Stegmann in 1985 [7]. As a nonperforating, blebless surgical technique, canaloplasty was first performed by Lewis et al. in 2007 [8]. It aims at restoring natural aqueous outflow by means of Schlemm's canal dilation which is achieved by tensioning with a 10.0 polypropylene suture. Canaloplasty has aroused substantial interest among surgeons over the last few years.

## 2. Patient Selection

Correct patient selection is the key to canaloplasty success. Canaloplasty is indicated for patients with mild-to-moderate primary open-angle glaucoma and a low-to-mid-IOP target [9]. Best indications are primary open-angle glaucoma, pseudoexfoliation glaucoma, and pigmentary glaucoma. Canaloplasty is also indicated for patients with advanced glaucoma who are not candidates for trabeculectomy. It may be recommended for patients with thin conjunctiva who could be at risk of bleb leaks and for individuals who cannot comply with the posttrabeculectomy care schedule [10]. After failed trabeculectomy, canaloplasty may be successful in patients with an undamaged Schlemm's canal. Counterindications to canaloplasty are conditions with a trabecular meshwork obstruction that prevents adequate cannulation of Schlemm's canal. These include chronic angle closure, narrow angles, angle recession, neovascular glaucoma, ocular hypertension due to increased episcleral venous pressure and previous surgery that precludes Schlemm's canal cannulation such as trabeculectomy, trabeculotomy, goniotomy, and argon laser trabeculoplasty [11].

## 3. Surgical Procedure

**Figure 1:**
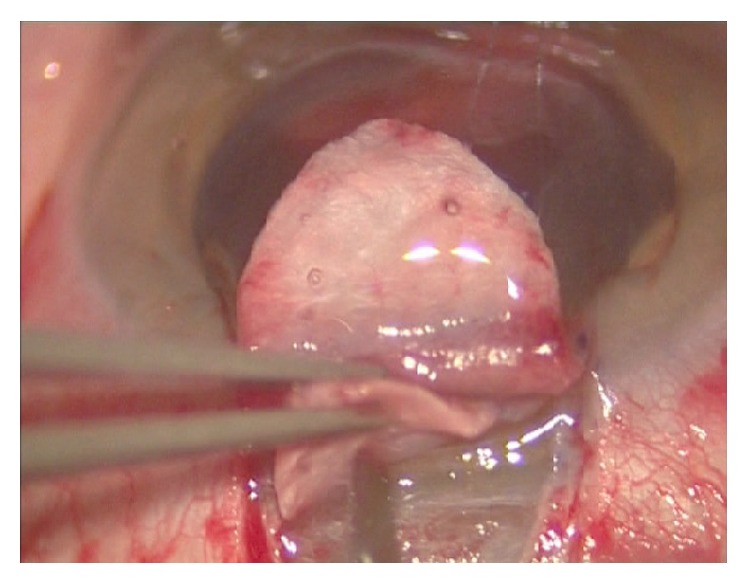


**Figure 2:**
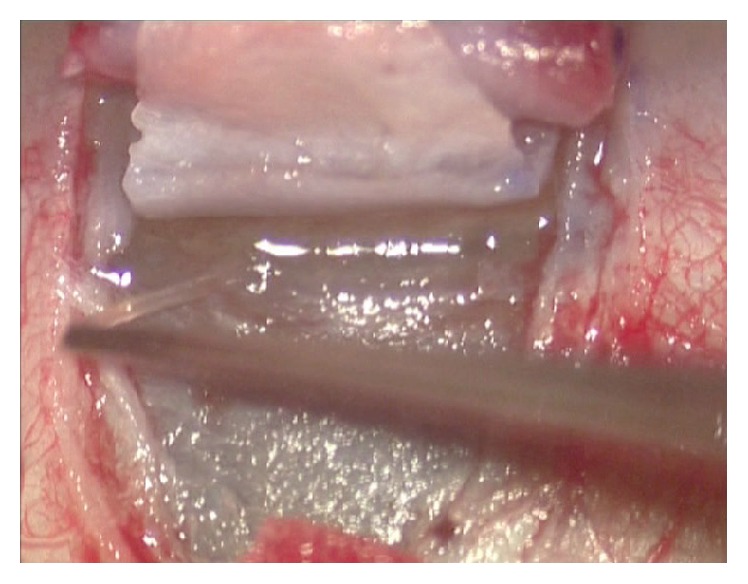


**Figure 3:**
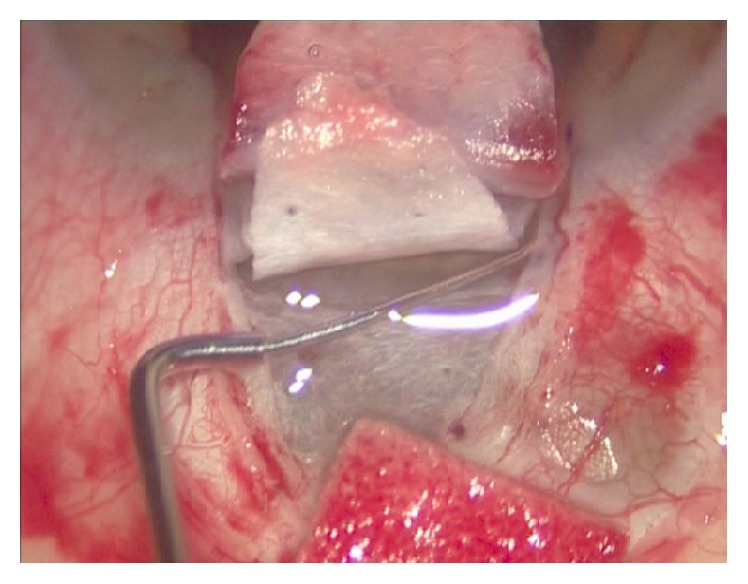


**Figure 4:**
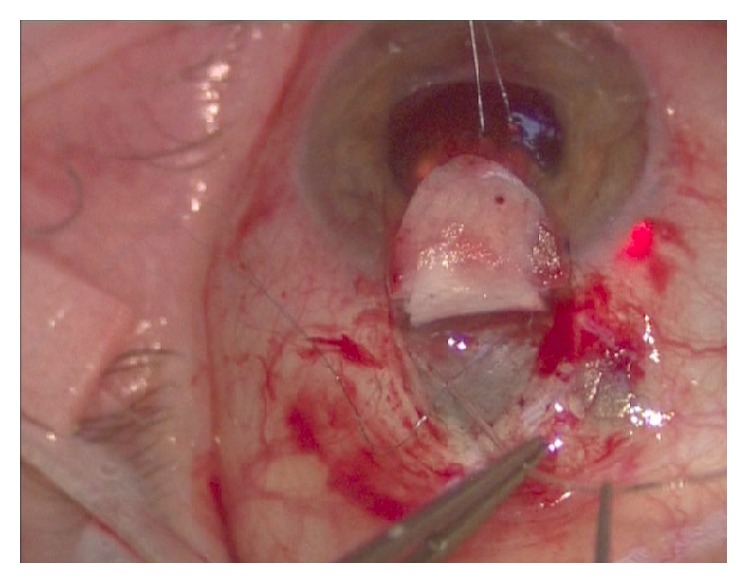


Canaloplasty is an ab externo procedure. A 10-0 polypropylene suture is positioned within Schlemm's canal for 360° and then tensioned to dilate the canal and restore natural aqueous outflow. Canaloplasty is usually performed in the upper quadrants, with access through the upper-temporal or upper-nasal quadrants. Some surgeons do, however, perform surgery in the lower quadrant. Although a retrobulbar block is commonly used, subconjunctival and topical anaesthesia is sometimes preferred. The eye is infero-ducted by placing a clear corneal traction suture near the limbus, and surgery starts by dissecting the fornix-based conjunctival flap. Careful conjunctiva and Tenon's capsule dissection should be followed by wet-field cautery. Antimetabolites are not used. Then, a nonpenetrating double-flap dissection of the sclera exposes Schlemm's canal. First, a square-shaped, triangular, or parabolic superficial scleral flap is created. Approximately one-third to half-scleral thickness, it is 5 mm wide and 5 mm long. This flap must be dissected up to the clear cornea about 1.5/2.0 mm over the limbus. Second, a deep scleral flap is dissected. A little smaller than superficial flap (one-half to one millimeter), its dissection plane should be immediately superficial to choroid. The deep scleral flap is dissected until ciliary body/choroid becomes visible (Figure 1) and Schlemm's canal is opened and deroofed by removing its inner wall (Figure 2). A paracentesis is then performed to lower IOP and reduce the risk of perforating the trabecular-Descemet membrane. The deep scleral flap is removed. Schlemm's canal openings are carefully dissected. One opening is cannulated using a flexible microcatheter (iTrack, iScience Interventional, Menlo Park, CA, USA) (Figure 3) which is pushed forward through the entire circumference of Schlemm's canal until it exits the other end. The microcatheter is 200 microns in diameter and its tip is illuminated by a laser-diode microillumination system (iLumin by iScience Interventional, Menlo Park, CA, USA) which easily identifies the distal tip through the sclera as it advances in the canal. When the microcatheter exits the opposite end, it is tied to a 10-0 polypropylene suture and is then withdrawn, by pulling it back through the canal in the opposite direction (Figure 4). During withdrawal, a dedicated injector inserts a small amount of viscoelastic material into Schlemm's canal every two hours. When the microcatheter encounters a stop the surgeon should not force the viscoelastic material any further because Schlemm's canal rupture could cause collector channel blood to pool in the Descemet detachment, increasing the risk of intracorneal hematoma formation.

When the suture arrives at the original end, it is cut from the microcatheter and the two ends are carefully tightened to pull the trabecular meshwork inwards. Achieving correct suture tension is crucial for success. Adequate contraction is needed for homogeneous stretching of the entire circumference but excessive contraction could break the inner wall (Figure 5). Correct suture positioning and canal tension are checked intraoperatively by means of a high-resolution ultrasound system (iUltrasound, iScience Interventional, Menlo Park, CA, USA). After deep scleral flap excision, the superficial scleral flap is closed tightly with 10-0 vicryl (or nylon) sutures. Watertight closure is essential to prevent bleb formation. The conjunctival flap is then sutured with 10-0 vicryl sutures [9, 12].

One of the major difficulties in canaloplasty is the tension suture placement and tightening. A flexible stent, the Stageman Canal Expander (SCE) (Ophthalmos GmbH, Schaffhausen, Switzerland) was developed to overcome this problem and to make canaloplasty easier. This 9.0 mm flexible stent is made of polyamide and is designed to fill a quarter of Schlemm's canal circumference, thus maintaining dilation and allowing the aqueous humor to access collector channels through its fenestrations. Using a 6/0 carrier, stents are placed in Schlemm's canal after microcatheter dilatation. When the microcatheter is withdrawn, stents are placed inside both ends. Stents overcome the obstacle of angles over Schlemm's canal that the polypropylene prolene tip finds difficult to pass over [12, 13].

## 4. Postoperative Treatment

Postoperatively, patients usually are treated with third or fourth generation fluoroquinolone drops 4 times daily for 1 week and with steroid drops 4 times daily for 1-2 weeks. Steroid therapy is generally tapered in the following 15–30 days. Some authors also administer nonsteroid anti-inflammatory drops in the first month of therapy [9].

## 5. Mechanism of Action

How canaloplasty lowers IOP is not fully understood but enlargement of Schlemm's canal and collector channels probably plays a role. In fact, canaloplasty is less likely to be successful in eyes with a nonreversible collapse of collector channels or other outflow pathways that cannot be mechanically enlarged. Since the effect of canaloplasty on IOP appears to be correlated, at least in part, to suture tension, some authors tried to measure the suture stent distension of Schlemm's canal inner wall. In a multicenter study using high-resolution anterior segment ultrasound biomicroscopy (UBM), Lewis et al. assessed the relationship between IOP reduction and the degree of canal distension. A grading system of suture tension on Schlemm's canal was created and eyes with a discernable postoperative distension were identified (first group) and compared to eyes without (second group). After two years, IOP was reduced by 31% in the first group and by 20% in the second [14]. Using UBM and anterior segment optical coherence tomography, Brandao et al. [15] quantified Schlemm's canal distension preoperatively and at 12 and 36 months after surgery. Both methods were equally efficient in identifying the suture/stent generated inner wall which did not change significantly over time. Moreover, when pre- and postoperative IOP differences were large, there was a tendency towards a greater Schlemm's canal distension, suggesting the tensioning suture contributed to IOP reduction. An adjunctive mechanism for IOP decrease could be enhanced aqueous humor filtration across the sclera and conjunctiva. Indeed, after successful canaloplasty, confocal laser-scanning microscopy demonstrated an increase in conjunctival microcysts, which are a sign of enhanced aqueous humor filtration across the sclera and conjunctiva [16].

## 6. Results

Reports concurred that canaloplasty effectively achieved good short- and long- term IOP reductions.

In 2007, Lewis et al. [8] published the results of a 1-year international multicenter prospective study, showing that canaloplasty reduced IOP by 36%. In 2009, the same authors [14] presented a 2-year follow-up of patients after canaloplasty, finding a 30% decrease in IOP (from 23.2 ± 4.0 to 16.3 ± 3.7 mm Hg). Need for medications also dropped from 2.0 ± 0.8 to 0.6 ± 0.8. Grieshaber et al. [17] published results of canaloplasty in 32 Caucasian subjects, observing that, without medications, mean IOP fell from 27 ± 5.6 to 12.8 ± 1.5 mm Hg 12 months after surgery. Complete success (IOP < 21 mm Hg without medications) was achieved in 93.8% of patients, IOP < 18 mm Hg in 84.4% of cases, and IOP < 16 mm Hg in 74.9%.

Grieshaber et al. [18] performed a long-term analysis in 2010 of canaloplasty outcomes in 60 black Africans with a mean preoperative IOP of 45 ± 12.1 mm Hg. At 3 years, with a mean follow-up of 30.6 ± 8.4 months, the mean IOP without medications was 13.3 ± 1.7 mm Hg. Complete success (IOP < 21 mm Hg without medications) was reached in 77.5% of patients and partial success (IOP < 21 mm Hg with or without medications) in 81.6%. In 2011, Lewis et al. [19] reported the results of a 3-year international multicenter prospective study on canaloplasty, as performed on 157 eyes with open-angle glaucoma. Preoperatively, mean IOP was 23.8 ± 5 mm Hg and number of medications was 1.8 ± 0.9. After three years, IOP decreased to 15.2 ± 3.5 mm Hg and the number of medications fell to 0.8 ± 0.9, with 36.1% IOP reduction from baseline. Complete success (IOP < 18 mm Hg without medications) was achieved in 36% of patients and partial success (IOP < 18 mm Hg with or without medications) in 77.5% [19].

In 2011, Bull et al. [20] analyzed the efficacy of canaloplasty in European patients with open-angle glaucoma, reporting 3-year outcomes in a series of 93 eyes. Mean IOP dropped from 23.0 ± 4.3 to 15.1 ± 3.1 mm Hg and the number of medications fell from 1.9 ± 0.7 to 0.9 ± 0.9. In 2014, Borisuth et al. [10] reported 3-year canaloplasty results in 214 eyes with open-angle glaucoma in patients who were under maximum medical therapy before surgery. The mean preoperative IOP of 29.4 ± 7.9 mm Hg decreased to 17.0 ± 4.2 mm Hg (42.2% mean IOP reduction). Complete success (defined as a postoperative IOP ≤ 21 mm Hg, ≤18 mm Hg and ≤16 mm Hg without any medical treatment) was achieved in 44.8%, 31.0%, and 24.1% of patients, respectively, while partial success (defined as a postoperative IOP ≤ 21 mm Hg, ≤18 mm Hg, and ≤16 mm Hg with or without medical treatment) was achieved in 86.2%, 58.6%, and 37.9% of patients, respectively.

In 2015, Voykov et al. [21] reported a 5-year follow-up on canaloplasty, observing the IOP reduction rate was similar to the 3-year rate. At 1, 3, and 5 years, complete success rates for IOP < 21 mm Hg were, respectively, 37%, 28%, and 10%, which are lower than the reported 40%–45% after three years. The low 5-year complete success rate could be a sign of canaloplasty losing efficacy over time. In this series, 65% of eyes required further surgery for IOP control.

Comparative studies showed canaloplasty was more efficacious in reducing IOP than viscocanalostomy [22] but less so than trabeculectomy with mitomycin C [23–25]. In a 1-year follow-up study, Ayyala et al. reported the mean IOP reduction was 32% after canaloplasty and 43% after trabeculectomy with mitomycin C; furthermore, postoperative need for medical therapy was 36% in the canaloplasty group and 20% in the trabeculectomy group [23].

Brüggemann et al. performed another comparative study on 30 eyes in 15 patients. They had trabeculectomy with mitomycin C in one eye (group 1) and canaloplasty in the other (group 2). After 1 year, mean IOP decreased from 26.3 ± 10.9 to 11.6 ± 5.2 mm Hg in group 1 and from 26.8 ± 6.4 to 13.2 ± 2.8 mm Hg in group 2. The number of glaucoma medications fell from 2.7 to 0.4 ± 0.7 in group 1 and from 2.5 to no medication in group 2. Moreover, the trabeculectomy group required a longer postoperative hospital stay (10.4 ± 2.8 versus 5.4 ± 1.0 days) as well as more postoperative check-ups (8.5 ± 3.6 versus 3.9 ± 0.8) and interventions [24].

In 2014, in a retrospective study, Thederan et al. [26] compared outcomes in 22 eyes after trabeculectomy and in 22 eyes after canaloplasty. Mean IOP in the trabeculectomy and canaloplasty groups decreased from 23.9 ± 10.7 to 10.8 ± 3.7 mm Hg and from 23.7 ± 7.6 to 14.5 ± 3.8 mm Hg, respectively. Complete success (defined as IOP < 21 mm Hg and 20* *% IOP reduction from baseline without medication), was achieved in 18 eyes (81.8* *%) after trabeculectomy and in 11 eyes (50.0* *%) after canaloplasty [26]. In a prospective, 2-year follow-up randomized clinical trial, Matlach et al. compared outcomes of canaloplasty and trabeculectomy in open-angle glaucoma. They observed that both approaches significantly reduced IOP. The mean absolute IOP reduction was 10.8 ± 6.9 mm Hg after trabeculectomy and 9.3 ± 5.7 mm Hg after canaloplasty while the mean IOP was 11.5 ± 3.4 mm Hg after trabeculectomy and 14.4 ± 4.2 mm Hg after canaloplasty. Complete success (IOP ≤ 18 mm Hg without medication) was achieved in, respectively, 74.2% and 39.1%, while partial success (IOP ≤ 18 mm Hg with or without medication) was achieved in 67.7% after trabeculectomy and in 39.1% after canaloplasty. Following trabeculectomy, complications were more frequent and included hypotony (37.5%), choroidal detachment (12.5%) and elevated IOP (25.0%) [25].

In 2014, Klink and coworkers [27] assessed quality of life and patient satisfaction after canaloplasty and trabeculectomy. Patients reported a better quality of life after canaloplasty, particularly with regard to positive postoperative mood, satisfaction with outcome, and lower rates of visual and nonvisual symptoms. In the trabeculectomy group, the authors registered higher stress rates due to surgery, postsurgical treatments, and check-ups and a lower satisfaction rate. They reported that 41% of patients were highly satisfied after trabeculectomy and 57% after canaloplasty. No intergroup differences emerged in restriction from social contacts and loss of independence.

## 7. Combined Surgery

Combining canaloplasty and phacoemulsification surgery is a suitable option in patients with clinically significant cataract. Cataract surgery alone is known to reduce IOP but the effect is generally small, probably because the wider angle and trabecular meshwork tensioning increased aqueous humor outflow [28]. The combined approach provided a greater hypotensive effect than canaloplasty alone [19, 29]. Angle architecture modification probably resulted in a more open configuration and in trabecular meshwork tensioning which increased aqueous humor outflow [28, 29].

In 2009, Lewis et al. [14] presented a 2-year follow-up of patients after phacocanaloplasty, finding a 42% decrease in IOP. IOP dropped from 23.1 ± 5.5 to 13.4 ± 4.0 mm Hg and the number of medications fell from 1.7 ± 1.0 to 0.2 ± 0.4. In a 3-year study after phacocanaloplasty, Lewis et al. found a 42.1% reduction in IOP from baseline. The mean IOP decreased from 23.5 ± 5.2 mm Hg with 1.5 ± 1.0 medications to 13.6 ± 3.6 mm Hg with 0.3 ± 0.5 medications. Use of hypotensive medications was reduced in 80% of patients [19]. In 2011, Bull et al. analyzed the efficacy of phacocanaloplasty in 16 eyes. The mean preoperative IOP fell from 24.3 ± 6.0 mm Hg with 1.5 ± 1.2 medications to 13.8 ± 3.2 mm Hg with 0.5 ± 0.7 medications after 3 years [20].

Similar results were found by Matlach et al. [30] 12 months after surgery. Phacotrabeculectomy lowered IOP more than phacocanaloplasty, but the difference was not significant. Moreover, the phacotrabeculectomy group needed fewer glaucoma medications (0.2 ± 0.4) than the phacocanaloplasty group (1.0 ± 1.5). In 2015, Schoenberg et al. [31] observed that phacocanaloplasty and phacotrabeculectomy resulted in comparable mean IOP at 12 months and they reported significant reduction in IOP and improvement in visual acuity with comparable success rates.

In 2015, in a comparative, prospective, randomized study, Rękas et al. [32] reported 1-year outcomes after phacocanaloplasty and phaco-nonpenetrating deep sclerectomy. Both techniques effectively reduced IOP with similar efficacy and safety profiles. Patients who underwent phaco-nonpenetrating deep sclerectomy required additional procedures like 5-FU injections, suture lysis, or needling, while phacocanaloplasty patients required no additional procedures.

## 8. Adverse Events

Although the rate of complications, particularly severe complications, is lower after canaloplasty than trabeculectomy [23], surgeons need to be aware of potential pitfalls that can occur during and after canaloplasty. Inability to cannulate Schlemm's canal is a major intraoperative complication as cannulation is successful in only 74–89.9% of cases [9, 19, 20, 29]. Failures may be due to anatomical anomalies of Schlemm's canal, to trabecular meshwork scars due to previous argon laser trabeculoplasty, or to surgical inexperience [19, 29]. When cannulation fails, the operation can be converted to deep sclerectomy or to viscocanalostomy [9].

Microcatheter escape from Schlemm's canal into surrounding structures is a rare complication. Grieshaber et al. reported catheter penetration of the anterior chamber in one case and of the suprachoroidal space in another [18]. These two cases accounted for 3.3% of all complications in the Grieshaber study.

Descemet's membrane detachment in 1.6–9.1% of cases is another rare intraoperative complication [9, 17, 19, 20, 29]. It may occur if, upon encountering a difficulty in cannulation, attempts to force injection of viscoelastic material lead to channel rupture [9]. Descemet's membrane detachments are usually limited in size (1-2 mm) and resolve spontaneously, but sometime they may reach the visual axis and require surgery [33].

Postoperative complications are divided into early (1–10 days after surgery) and late (2–5 weeks after surgery). The most common early postoperative complication, which is observed in 6.1% to 85.2% of cases, is bleeding from Schlemm's canal into the anterior chamber on the first day after surgery [9, 19, 20, 34]. Blood reaching the anterior chamber from the collector channels is considered a positive prognostic factor because it may indicate the outflow pathways are open and functioning [34]. Hyphema is usually transient, resolving spontaneously and without consequences in up to one month [17, 19, 20].

A transient increase in IOP that can reach 30 mm Hg or more is another early postoperative complication that was reported in 1.6–18.2% of eyes. It is probably due to residual viscoelastic material in Schlemm's canal that prevents aqueous humor from passing into the collector channels. IOP usually stabilizes after 24–48 hours when all residual viscoelastic is reabsorbed [9] and the increase rarely persists for 3-4 weeks. Should it do so, laser goniopuncture might be efficient.

Hypotony, a rare, transient complication (0.6%–9.8%) [9, 19, 20], is due to superficial flap sutures not being watertight.

Late complications of canaloplasty include cataract formation in 12.7% to 19.1% of patients according to diverse studies [19, 20] and long-term failure which may be treated with laser goniopuncture to reduce IOP and, if that fails, trabeculectomy.

Bleb formation is rare (2.5%) especially after phacocanaloplasty [19, 35].

## 9. Conclusions

Canaloplasty appears to be a valid alternative to conventional glaucoma surgery because of its efficacy and safety profile in selected patients with open-angle glaucoma. Its advantages over trabeculectomy include absence of subconjunctival bleb, no need for mitomycin C, faster visual rehabilitation, easier follow-up, and fewer postoperative complications. On the other hand, disadvantages are the long learning curve, need for specifically designed instruments, impossibility to perform the surgery properly in some cases, and a lower IOP reduction compared with trabeculectomy.

## Figures and Tables

**Figure 5:**